# New Instrument Designed to Remedy the Imperfections of Speech Consequent upon Congenital Fissure of the Soft Palate

**Published:** 1847-04

**Authors:** C. H. Stearns

**Affiliations:** Surgeon; 2 Vernon-place, Bloomsbury


8. *A New Instrument, Designed to Remedy the Imperfections of Speech Consequent upon congenital Fissure of the Soft Palate.*—By C. H. Stearns, Esq., Surgeon.

As the readers of the Lancet are doubtless well acquainted with the means, both surgical and mechanical, which have hitherto been employed in cases of fissure of the palate, with the hope of improving the articulation, no review of the subject is here deemed necessary. A near relation of the writer of this communication had twice undergone the operation of staphyloraphy, and had also submitted himself several times to the hands of dentists, who professed to be able to close up the fissure by the adaptation of mechanical contrivances. These measures not being attended with the slighest benefit, the writer was induced himself to attempt something for his relief; and at length concieved the plan of an instrument, which from its proposed shape, position, and mobility, seemed likely to perform, to some extent at least, the functions of the natural *velum palati*, or soft palate. After a length of time, a piece of mechanism was produced, the application of which has been attended with satisfactory results. As it is probable that something of the kind may prove equally useful in other cases, a brief description of the affair is here offered.

A gold plate is first fitted to the roof of the mouth, in the manner practiced by dentists, which is to serve as the foundation or support of the mechanism intended to supply the want of the natural soft palate. To the upper and posterior margin of this plate, a flat spiral spring is attached, which, with the delicate and permanent elasticity peculiar to that kind of spring, admits of easy and constant vibrations backwards and forwards. To the other and posterior extremity of this spring, an artificial *flexible* velum is attached. This part of the instrument is constructed of Mr. Goodyeare's preparation of caoutchouc, which, having the property to resist the action of both oils and acids, and at the same time sustaining a high degree of heat, has proved well adapted to the purpose. In attempting to describe the artificial velum, we must, for the want of better terms at present, designate its principal parts as its *body and wings.* The body of the velum consists of the lamina of the caoutchouc, of a somewhat triangular form, and of the same size and shape as the vacant space is intended to occupy, that being the plane which would be indicated by imaginary lines connecting the opposite side or columns, and subtending the vertical angle of the fissure, at which point the velum is connected to the posterior extremity of the spiral spring. This lamina, constituting the body of the velum, is divided into three pieces which overlap each other. The wings projected obliquely forwards and outwards from each lateral margin of the body, and being made to conform to the shape of the columns or sides of the fissure, are seen to rest upon their inner and anterior surfaces, thus covering a portion of the soft parts which constitute the boundaries of the posterior fauces. In like manner, along each lateral margin of the body, there is (in mechanical phrase) a flange, projecting obliquely backward and outwards, and extending along down the posterior surface of the column, it terminates at the inferior angle of the velum. In this way the wing and the flange, on the same side, together form a groove fitted to receive the fleshy sides of the fissure. As the preparation of caoutchouc made use of presents a smooth surface, and yields readily to the slightest pressure, it is found to permit the contact and muscular motion of the surrounding soft parts, without causing any irritation. When, therefore, the sides of the fissure tend to approximate, as in deglutition, gargling the throat, or the utterance of some of the short vowel sounds, the three parts of the velum slide readily by each other, thus diminishing the extent of the exposed surface, and thereby imitating, to some extent, muscular contractile action, the force being derived from without, and not, of course, contained within the instrument. During the effort made in speaking, the surrounding muscular parts embrace and close upon the artificial velum, and press it back against the concave surface of the pharynx. The passage to the nares being therefore temporarily closed, the occlusion of sound is accomplished, and articulation made attainable, as the voice or sound, as it issues from the glottis, is thereby directed into the cavity of the fauces, and confined there long enough to receive the impressions made upon it by the tongue, lips, &c., in the formation of consonant letters.

The foregoing description may not be thought sufficiently specific; but some considerations preclude, at the present time, a more detailed account, which, to be intelligible, would require the aid of figures to illustrate the mechanism of the instrument. Even that might fail to satisfy one much interested in the subject, without an opportunity being offered of witnessing actual results derived from its application.

Though the instrument, after having been adapted in the way above described, was found materially to improve the speech, yet it was still considered defective, and not admitting of general application, until other important requisites had also been attained; for it was necessary to make it so yielding as not to irritate the sensitive and restless parts with which it must come in contact; so that it might at all times be retained in place without inconvenience, while eating, drinking, or during sleep. At the same time it was required to possess a degree of strength and firmness sufficient to sustain the force of any sudden shock, as in coughing, sneezing, or laughing, without the risk of being displaced, or in any way deranged. Durability of the substance composing the velum was also regarded as a point of the first importance to ensure its usefulness. The material made use of, as prepared by Mr. Goodyeare, and managed according to his instructions, was found (after some practice in the manipulation necessary to bring it to the shape required), to resist the combined action of all the decomposing agents to which it must become subjected—viz.: motion, animal heat, the moisture and acids of the mouth, and the oils of the food. The means afterwards devised to keep it in order, freeing it from deposits, and thus preventing fetor, consist in the occasional use of some alkaline or aromatic preparation.

Any one who has had an opportunity of seeing many cases of congenital fissure of the palate, must have observed that they present considerable diversity in their anatomical features. To meet the peculiarities of each case, therefore, renders a corresponding modification of the metallic part of the instrument necessary; but the same method of constructing the artificial velum is applicable to all the varieties the writer has yet met with. In those cases where, as in persons with hare-lip, the fissure extends quite through the palatine and superior maxillary bones; the gold plate employed to sustain the velum will of course complete the defective part of the roof of the mouth.

We would now willingly add some account of the elocutionary practice and discipline resorted to, in order to obtain the full benefit of the instrument after its adaptation; but this may well be deferred to a future paper; more space having already been occupied than was at first intended—the purpose of this communication is indeed merely to announce what had thus far been accomplished.

2 *Vernon-place*, *Bloomsbury*, *June*, 1845.

Note.—We are happy to give publicity to Mr. Stearns' very ingenious invention, which we really believe calculated to relieve a most distressing infirmity.

We have seen the instrument applied in a well-marked case of congenital fissure of the velum palati, and found Mr. Stearns' promises fully substantiated. The articulation, previously very imperfect, at once became so natural that a person not acquainted with the patient would scarcely have imagined there was any defect whatever in the organs of speech.—Ed. Lancet.—Lond. Lancet in *Amer Jour. of Dent. Sci.*

